# Supplementary material for: Patterns of Intron Gain and Loss in Fungi
Source: PLoS Biol. 2004 Nov 30;2(12):e422. doi: 10.1371/journal.pbio.0020422 (PMC532390; doi:10.1371/journal.pbio.0020422)
Supplement: Table S1 — Also available at http://genes.mit.edu/NielsenEtAl/. (4.3 MB ZIP). [file pbio.0020422.st001.zip › NielsenEtAl/html/600.html]

AN6688.1.NCU08297.1.MG01521.1.FG05315.1


```
 CLUSTAL W (1.82) Multiple Sequence Alignments - Introns Inserted


Sequence 1: NCU08297.1	398 aa
Sequence 2: MG01521.1	388 aa
Sequence 3: FG05315.1	388 aa
Sequence 4: AN6688.1	405 aa
Alignment Length: 406 aa
Number Identitical Residues: 333 aa
Alignment Score (without introns) 13278


MG01521.1 	-----------------MPNQWHRKSVRKGFNFNVMVVG1ESGLGKSTLVNTLFNTSLYP
NCU08297.1	--------MTGYVGFANLPNQWHRKSVRKGFNFNVMVVG1ESGLGKSTLVNTLFNTSLYP
FG05315.1 	-----------------MPNQWHRKSVRKGFNFNVMVVG1ESGLGKSTLVNTLFNTSLYP
AN6688.1  	MKNVVRRKLTGYVGFANLPNQWHRKSVRKGFNFNVMVVG1ESGLGKSTLVNTLFNTSLYP
          	 ..    . :.  . :.:********************* ********************

MG01521.1 	PKEQKGPSLDIIPKTVTIQSISADIEEAGVRLRLTVVDTPGFGDFVNNDESWRPITDNIE
NCU08297.1	PKERKGPSLDIVPKTVTIQSISADIEEAGVRLRLTVVDTPGFGDFVNNDESWRPIVDNIE
FG05315.1 	PKERKGPSLDIIPKTVTIQSISADIEEAGVRLRLTVVDTPGFGDFVNNDESWRPIVDNIE
AN6688.1  	PKERAGPSHDIIPKTVSIQSISADIEENGVRLRLTVVDTPGFGDFVNNDDSWRPIVENIE
          	***: *** **:****:********** *********************:*****.:***

MG01521.1 	QRFDAYLDAENKVNRVNIVDNRIHACVFFIQPTGHSLKPLDIEVMKRLHTKVNLIPVIAK
NCU08297.1	QRFDAYLDAENKVNRMNIVDNRIHACVFFIQPTGHSLKPLDIEVMKRLHTKVNLIPVIAK
FG05315.1 	QRFDSYLDAENKVNRMNIVDNRIHACVFFIQPTGHSLKPLDIEVMRRLHTKVNLIPVIAK
AN6688.1  	QRYDAYLEAENKVNRTNIIDNRIHACVYFIQPTGHSLKPLDIEVMRRLHTKVNLIPVIAK
          	**:*:**:******* **:********:*****************:**************

MG01521.1 	SDTLTDEEIFAFKQR0ILADIKHHKVQIFEGPRYELDDEETIAENNEIMSKVPFAVVGAT
NCU08297.1	SDTLTDDEIAAFKAR0ILADIKYHKVQIFEGPRYELDDEETIAENNEIMSKVPFAVVGAT
FG05315.1 	ADTLTDEEIAAFKSR0ILADIKHHGIQIFEGPRYELDDEETIAENNEIMSKVPFAVVGAN
AN6688.1  	ADTLTDDEISMFKKR0ILADIQHHSIQIFEGPRYELDDEETIAENQEIMSKVPFAVVGAN
          	:*****:**  ** * *****::* :*******************:*************.

MG01521.1 	NEITNADGRKVRGRRYPWGVIEVDNEEHCDFVKLRQMLIRTHMEELKEHTNNILYENYRT
NCU08297.1	NEITNADGRKVRGRRYPWGVIEVDNEEHCDFVKLRQMLIRTHMEELKEHTNNVLYENYRT
FG05315.1 	NEITSADGRKIRGRAYPWGIIEVDNEEHCDFVKLRQMLIRTHMEELKEHTNNQLYENYRT
AN6688.1  	SEVATSDGRKVRGRSYPWGIIEVDNEEHCDFVKLRQMLIRTHMEELKEHTNNHLYENYRS
          	.*::.:****:*** ****:******************************** ******:

MG01521.1 	DKLIQMGVSQDPSVFKEVNPAVKQEEERTLHEQKLAKMEAEMKLVFQQKVAEKESKLKQS
NCU08297.1	DKLIAMGVSQDPSVFKEVNPAVKQEEERALHEQKLAKMEAEMKMVFQQKVAEKESKLKQS
FG05315.1 	DKLLAMGVSQDPSVFKEVNPAVKQEEERALHEQKLAKMEAEMKMVFQQKVAEKESKLKQS
AN6688.1  	DKLTQMGVAQDPSVFKEVNPAVKQEEERALHEQKLAKMEAEMKMVFQQKVAEKESKLKQS
          	***  ***:*******************:**************:****************

MG01521.1 	EEELYTRHREMKEQLDRQRAELEEKKARVESGRPLEKEP-KRKGFSLR
NCU08297.1	EEELYARHREMKEQLERQRLELEEKKSRIESGRPIEKEPTKRKGFSLR
FG05315.1 	EEELYARHKEMKEQLDRQRLELEDKKQRVESGRPLEKEG-KRKGFSLR
AN6688.1  	EDELYARHREMKEQLDRQRAELEEKKARLESGRPLEEKG-KRKGFSLR
          	*:***:**:******:*** ***:** *:*****:*::  ********
```
